# VATS Lobectomy: Surgical Evolution from Conventional VATS to Uniportal Approach

**DOI:** 10.1100/2012/780842

**Published:** 2012-12-30

**Authors:** Diego Gonzalez-Rivas

**Affiliations:** Department of Thoracic Surgery, Coruna University Hospital and Minimally Invasive Thoracic Surgery Unit, 15006 Coruna, Spain

## Abstract

There is no standardized technique for the VATS lobectomy, though most centres use 2 ports and add a utility incision. However, the procedure can be performed by eliminating the two small ports and using only the utility incision with similar outcomes. Since 2010, when the uniportal approach was introduced for major pulmonary resection, the technique has been spreading worldwide. The single-port technique provides a direct view to the target tissue. The conventional triple port triangulation creates a new optical plane with genesis of dihedral or torsional angle that is not favorable with standard two-dimension monitors. The parallel instrumentation achieved during single-port approach mimics inside the maneuvers performed during open surgery. Furthermore, it represents the less invasive approach possible, and avoiding the use of trocar, we minimize the compression of the intercostal nerve. Further development of new technologies like sealing devices for all vessels and fissure, robotic arms that open inside the thorax, and wireless cameras will facilitate the uniportal approach to become the standard surgical procedure for pulmonary resection in most thoracic departments.

## 1. Introduction

Video-assisted thoracoscopic surgery (VATS) lobectomy has become the treatment of choice for lung tumours. VATS lobectomy can be defined as the individual dissection of veins, arteries, and lung lobar bronchi, together with mediastinal lymphadenectomy, using a videothoracoscopic approach visualized on screen and involving 2 to 4 incisions or ports in the absence of rib spreading. It is important to distinguish it from hand-assisted resections and resections assisted via thoracoscopy that use a rib retractor and involve direct surgeon visualization of the surgical field. Some studies have demonstrated the advantages of the approach without rib spreading [1].

Although lobectomy must be performed anatomically, some authors such as Lewis and Caccavale presented twenty years ago the first 40 published reports of VATS lobectomy, with good outcomes via simultaneous stapled lobectomy [2]. The video-assisted thoracic surgery (VATS) anatomic lobectomy for lung cancer was initially described in 1992 [3, 4].

## 2. Evolving Technique

There is no standardized technique for the VATS approach, though most centres use a utility incision measuring about 3–5 cm and generally positioned anteriorly. Most surgeons then add two other ports (one for the optics and another at posterior level). Groups such as those led by Gossot et al. [5] or Mun and Kohno [6] describe purely thoracoscopic lobectomies involving three incisions with a minithoracotomy only for extraction of the lobe. Some Japanese groups describe the use of silicon rubber for separating the soft tissues in the utility incision, and for facilitating the bronchoplastic procedures [7]. Recently reported series also demonstrated good outcomes for fully thoracoscopic segmentectomies [8].

McKenna Jr et al., in his publication of 1100 cases [9], describes excellent results with three ports and occasionally a fourth port (percentage conversion 2.5%, with a median stay of 3 days). Using this type of approach the author has published 13 cases of sleeve resection [10].

The group led by Damico has the largest reported series to date in double-port VATS. These authors published a large series of 500 cases with a surgical conversion rate of 1.6% and a median hospital stay of three days [11]. They have recently published a series of 697 VATS lobectomies with fewer postoperative complications than when performing thoracotomy [12] and 48 thoracoscopic segmentectomies with good results [13].

Many articles in the literature suggest that VATS lung resection is associated with reduced postoperative pain when compared with conventional thoracotomy [14]. According to the review published by Coffey et al., lung resections performed on a minimally invasive basis could favourably influence oncological outcomes obtained [15]. Various studies have reported a lower acute phase inflammatory response, with a lower release of interleukins and C-reactive protein in minimally invasive procedures, with preservation of the host immune response [16, 17]. Likewise, as this is a minimally invasive procedure, early patient discharge is enhanced, with fast access to adjuvant chemotherapy [18]. The future growth of indications for VATS resection for lung cancer includes those patients who are at high risk, for example, older patients and those with poor pulmonary function [19].

## 3. Learning Curve

Some studies consider the learning curve for VATS lobectomy to comprise about 50 procedures [20]. We started to perform VATS lobectomies in our department in 2007 after learning the technique at Cedars Sinai, Los Angeles, CA, USA [9]. After performing over 80 lobectomies with three ports (3P), we eliminated the posterior incision and subsequently performed most lobectomies through only two ports (2P) according to the technique described by Burfeind and D'Amico [21]. In our initial series of patients, at the beginning of the program the mean duration of surgery was globally similar with either two or three ports (168.6 min in 2P versus 163.39 min in 3P). However, differences were observed on analyzing the results according to whether upper or lower lobectomies are involved. By obviating the posterior incision we lost a port for lung traction and the insertion of endostaplers. This initially led to a prolongation of surgery time in the case of upper lobectomies (199.3 min in 2P versus 169.04 min in 3P, *P* = 0.029). However, on examining the duration of upper lobectomy in the second period of 2P, we observed no significant differences versus the triple-port group. This seems to indicate that as more procedures were performed and surgeon experience with the double port procedure increases, times were shortened and become similar to those recorded with the triple-port approach. 

## 4. Mediastinal Lymph Node Dissection

A very important consideration is how mediastinal lymphadenectomy was performed without the third port entry—the latter understood as systematic nodal dissection. We currently perform routinely lymph node dissections in patients undergoing lobectomy for NSCLC via the VATS approach. The area posing the greatest difficulties is the left subcarinal region. Omitting the posterior port and performing complete lymphadenectomy from the utility incision may seem complicated, though with good technique and increased experience the outcomes are similar to those afforded by the triple-port approach as regards the number of lymph node stations and the number of adenopathies. When we compared our initial results of three- versus double-port technique, the number of lymph nodes resected in 3P was globally greater than in 2P (11.77 versus 9.23, *P* = 0.023). In contrast, the number of lymph node stations was similar in both groups. However, if we divided the time into two different periods, an early and a latter period, we observed that the latter period (when experience is gained) in 2P showed no significant differences versus the triple-port group as regards the global number of adenopathies and the number of left side adenopathies removed. As more cases were treated with the double-port approach, the number of lymph nodes removed increases, reflecting improvement in executing the surgical technique. In any case, we performed the adequate mediastinal staging proposed by some authors [22].

Many articles have found the results of VATS lymphadenectomy to be equivalent to those obtained via thoracotomy [23–26]. Japanese groups have described series almost all involving three or four incisions, generally with a larger utility incision (5–7 cm), and using a lap protector [27, 28]. Watanabe et al. concluded that complete lymphadenectomy can be performed with VATS without having to convert to open surgery even when stage N2 disease is confirmed intraoperatively [29]. We consider the important issue to be the observation of the oncological principles and to feel comfortable with the technique, in order to perform adequate mediastinal resection through two, three, or four ports—with conversion in those cases in which we are not sure that complete lymphadenectomy can be performed, particularly in the presence of stage N2 disease. The feasibility and safety of the procedure depends on the surgeon's experience.

## 5. The Single-Port Era

The surgical evolution in the approach from three-ports to double-port technique required a new learning curve: different lung exposure and learning how to move the camera from the camera port to the utility incision during surgery. But the final step of the surgical evolution in our unit, to minimize chest wall trauma, was the uniportal approach for major lung resections.

Since 2004, Rocco et al. have published different articles on the single-port VATS technique [30, 31] for diagnostic and therapeutic procedures, though not including lobectomies. We started to perform major pulmonary resections by uniportal approach in June 2010 [32]. All of the cases were performed by surgeons with previous experience in VATS surgery, specially in double-port technique for major pulmonary resections and single-port technique for minor procedures (wedge resections, pneumothorax, etc.). Initially, only lower lobes cases were selected [32, 33]. As with all new surgical procedures, there was a certain learning curve component but not comparable to the one experienced when starting a VATS program [20]. For double-port VATS lower lobectomies, all the instrumentation and stapler insertion were performed through the utility incision. Therefore we decided to insert the optic through the utility incision working in coordination with instruments to perform a single incision lobectomy. The first case we performed was accomplished in 90 minutes and the patient was discharged on the second postoperative day with no complications [32]. When several lower lobes cases were performed with good results, the upper lobes were attempted [34].

Single-incision VATS lobectomy follows the oncological principles of major pulmonary resections by VATS: individual dissection of veins, arteries, and lobar bronchus, likewise complete mediastinal lymphadenectomy with a video-assisted thoracoscopic approach, with no rib spreading [35]. The procedure is performed by video visualization. It is important to distinguish it from hand-assisted resections and resections assisted via thoracoscopy which use a rib retractor and involve direct visualization of the surgical field. Some studies have demonstrated the advantages of the approach without rib spreading [1].

**Figure 1:**
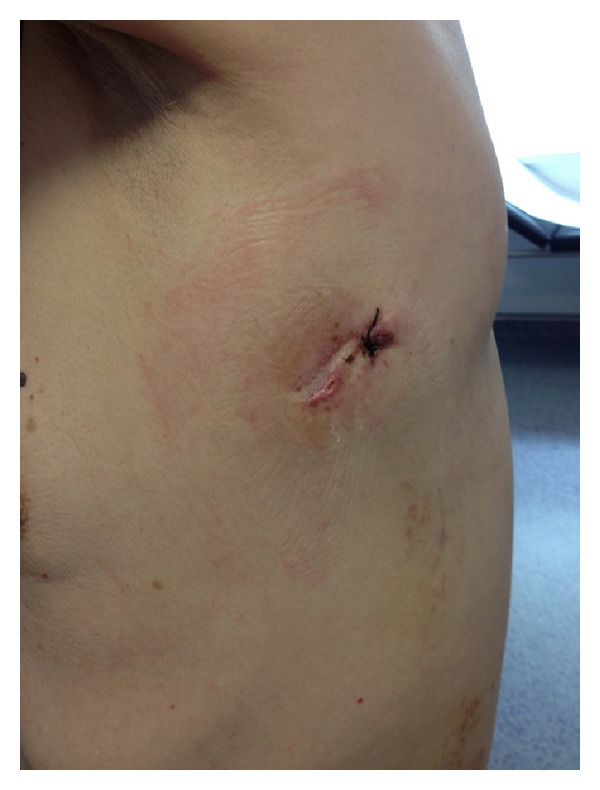
Postoperative result of single incision.

**Figure 2:**
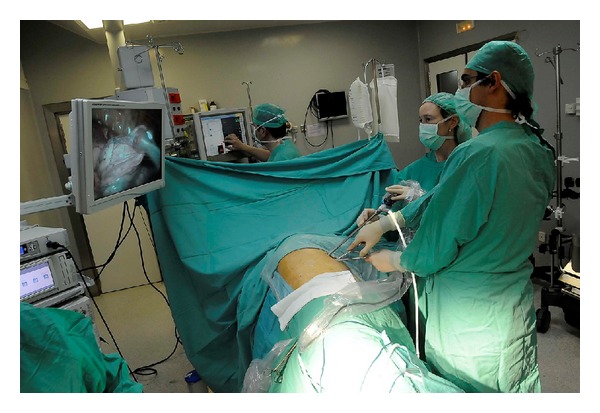
Surgeons in front of the patient.

**Figure 3:**
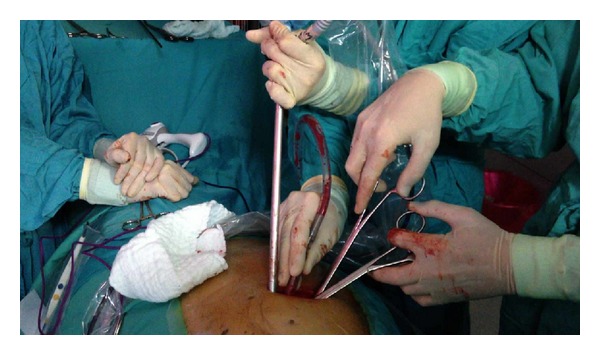
Surgical instrumentation.

The size of the utility incision is comparable to those commonly used for double- or triple-port approach [9, 11]. The incision is usually made at the level of the 5th intercostal space (Figure 1) to get good access to upper hilar structures and lymph node stations. The proper placement of this incision is crucial for good access to upper hilar structures and lymph node stations. Adequate exposure of the lung is mandatory for successfully completing the major resection. We are certain that this procedure could be performed more successfully in a center already using the double-port technique. The surgeon and the assistant must be placed in front of the patient in order to have the same thoracoscopic vision during all steps of the procedure and be more coordinated with the movements. (Figure 2). Instruments must preferably be long and curved to allow the insertion of 3 or 4 instruments simultaneously (Figure 3). Optimal exposure of the lung is key in order to facilitate the dissection of the structures and to avoid instrument malposition.

Even though the field of vision is only obtained through the anterior access site, the movement of the camera alongside the incision as well as its rotation will create different angles of vision (30 degree camera recommended to achieve a panoramic view). The advantage of using the camera in coordination with the instruments is that the vision is directed to the target tissue, bringing the instruments to address the target lesion from a straight perspective, thus we can obtain similar angle of view as for open surgery. Conventional three-port triangulation makes a forward motion of VATS camera to the vanishing point. This triangulation creates a new optical plane with genesis of dihedral or torsional angle that is not favorable with standard two-dimension monitors. Instruments inserted parallel to the videothoracoscope also mimic inside the chest maneuvers performed during open surgery. There is a physical and mathematical demonstration about better geometry obtained for instrumentation and view in the uniportal VATS over conventional approach.

Another potential advantage of the single port-approach could be a reduction in postoperative pain. There could be several explanations for this issue: only one intercostal space is involved and avoiding the use of a trocar could minimize the risk of intercostal nerve injury (during instrumentation, we try to apply the force over the superior aspect of the inferior rib through the utility incision). We have observed that patients operated by conventional VATS sometimes refer their pain towards the posterior and inferior incision and only a few times refer pain in the utility incision. We strongly believe that this pain could be explained by trocar compression over the intercostal nerve during camera movement. Some authors have reported less postoperative pain and fewer paresthesias in patients operated on for pneumothorax through a single incision, in comparison to the classical triple-port approach [36, 37]. Further studies will be required to demonstrate that there is less pain with single-incision techniques, compared to conventional VATS for lobectomy.

## 6. Single-Port Lobectomy: Early Results

Since July 2010, we have performed 134 uniportal VATS lobectomies. Only 6 patients were not completed by single port (4 conversions and 2 cases finished by double-port VATS). Previously, different groups have reported conversion rates for standard VATS lobectomy that range from 2% to 14% [38]. The low conversion rate for our single port lobectomies was achieved by experience gained from previous VATS surgery in vascular dissection, in management of fissures, in complex cases including postchemotherapy, and those with dense adhesions. The success in performing uniportal lobectomies is a consequence of skills and experience accumulated over time through VATS surgery. 

We believe this procedure should neither prolong operative time, nor hinder dissection or clearance of lymph nodes, nor increase the likelihood of surgical or postoperative complications compared with our double- or triple-port VATS techniques [39]. The mean operative time for our single-port lobectomies was 154.1 ± 46 minutes (range, 60–310 minutes), and the mean number of nodal stations explored in patients diagnosed with NSCLC was 4.7 ± 1.2 (1–8) with a mean of 14.7 ± 6.9 (5–38) lymph nodes resected.

In general, it is unusual for all unit members to master a new technique; however, we are fortunate that only within two years the 3 staff surgeons at our hospital are already routinely performing the uniportal VATS technique with good postoperative results. Furthermore, with increased experience, the technique is refined and taught to other surgeons in our unit, so that even early residents have performed uniportal lobectomies. At our unit, we started with lower lobes but soon extended the indications to upper lobes and advanced cases [40].

## 7. Conclusion

We truly believe in the use of the single-port technique for major pulmonary resections because we understand that the future goes in that direction, that is, robotics and single port. We expect further development of new technologies like sealing devices for all vessels and fissure, robotic arms that open inside the thorax, and wireless cameras, which will probably allow the single-port approach to become the standard surgical procedure for major pulmonary resections in most thoracic departments.
